# Prevalence and factors associated with teenage pregnancy in Sierra Leone: evidence from a nationally representative Demographic and Health Survey of 2019

**DOI:** 10.1186/s12889-023-15436-x

**Published:** 2023-03-20

**Authors:** Lilian Nuwabaine, Quraish Sserwanja, Kassim Kamara, Milton W. Musaba

**Affiliations:** 1 School of Nursing and Midwifery, Aga Khan University, 65 House No. 227, Kampala, Uganda; 2 Programmes Department, GOAL Global, Arkaweet Block 65 House No. 227, Khartoum, Sudan; 3 National Disease Surveillance Programme, Ministry of Health and Sanitation, Free town, Sierra Leone; 4 Department of Obstetrics and Gynaecology, Mbale Regional Referral and Teaching Hospital, Mbale, Uganda; 5 Department of Obstetrics and Gynaecology, Busitema University, Tororo, Uganda

**Keywords:** Teenage, Pregnancy, Sierra Leone, Adolescents, DHS

## Abstract

**Background:**

Globally, teenage pregnancy remains a public health concern because of the associated maternal and perinatal morbidity and mortality. To address the extensive social, political and economic effects of teenage pregnancy, there is need for current epidemiological evidence on its prevalence and associated factors, especially from low resource settings where the burden is highest.

**Methods:**

We used data from the 2019 Sierra Leone Demographic and Health Survey (SLDH), which included 3,427 female adolescents. Multistage stratified sampling was used to select study participants. Teenage pregnancy was defined as those who had ever either had a child, or terminated a pregnancy, or were currently pregnant. Multivariable logistic regression was conducted to determine the factors associated with teenage pregnancy using SPSS version 25(Armonk, NY: IBM Corp).

**Results:**

The prevalence of teenage pregnancy was 22.1% [758/3,427]. Of these, 17.8%, (608/3427), had ever had childbirth, 4.2%, (144/3427), were pregnant, and 1.2%, (40/3427) had ever terminated a pregnancy. After adjusting for confounders, the odds of teenage pregnancy among married girls were about 15 times more than the odds among those who were not married (aOR; 15.31, 95% CI: 11.17–20.98) while the odds of teenage pregnancy among girls from the poorest households were 2.5 times more than the odds among girls from the richest households.

**Conclusion:**

The prevalence of teenage pregnancy in Sierra Leone is high. To reduce teenage pregnancy, the government of Sierra Leone and its partners should target married, older teenagers and those from poor households. Policies giving teenage mothers a second chance by encouraging them to return to school after childbirth should be encouraged as an alternative to early marriages.

## Introduction

Adolescence is such a distinctive transitional period from childhood to adulthood that is often characterized by physical, psychological and social changes [1]. It is also an important time for these teenagers to lay the foundations of good health in adulthood [2]. Teenage, has been defined by the World Health Organization as the period from 10 to 19 years of age, and is generally classified into early adolescence (10–14 years) and late adolescence (15–19 years) [3, 4]. Recent reports indicate that over 16 million adolescent girls become pregnant globally every year, with 95% of these happening in low and middle income countries [5]. Although the prevalence of adolescent birth rates globally have fallen from 65 births per 1000 women in 1990 to 47 births per 1000 women in 2015, adolescent pregnancies have remained unacceptably high in sub-Saharan Africa [2, 6, 7], despite several efforts from government and non-government actors. A recent meta-analysis by Kassa et al. revealed that adolescent pregnancy stands at 18.8% in the whole of Africa, while the rate is at 19.3% in Sub-Saharan Africa, with rates ranging from 21.5% in East Africa to 9.2% in North Africa [8]. In some countries, the rates of teenage pregnancy are as high as 44.3% in Congo, 39.4% in Angola, 38% in Gabon, and 38.9% in Liberia [6].

Sierra Leone is not only ranked among the countries with the worst maternal and child health indicators globally [9]. About 16.8% of women in Sierra Leone have had sexual intercourse before age 15, 13.9% of all the women of reproductive age who are currently in a union are aged 15 to 19 years with this age group having the highest unmet need for family planning currently at 27.8% [10]. In addition, Sierra Leone is among the ten countries with the highest rates of teenage pregnancy in the world [9]. The reported rates of teenage pregnancy range from 28.3% by Ahinkorah et al.[6] to 34% by Bash-Taqi et al. [11], with an adolescent fertility rate standing at 102 per 1000 [10]. Of the reported 857 maternal deaths per 100,000 live births in Sierra Leone [12], an estimated 40% occur among teenagers [11].

Furthermore, Sierra Leone’s health system faces frequent stock outs of medical supplies, shortage of skilled and motivated health workers [13–16] making it hard to provide quality healthcare services especially to pregnant teenagers who are at high risk of maternal morbidity and mortality because of their social disadvantages. Therefore, there is need for urgent critical analysis of teenage pregnancy and associated factors using the most recent national data, if the country’s efforts of achieving Sustainable Development Goal (SDG) 3.1 that aims at global reduction of Maternal Mortality Ratio (MMR) to less than 70 per 100 000 live births by 2030 are to succeed [17, 18]. Studies have partly attributed the trends to the country’s post-conflict context, in which teenage girls face profound structural exclusion, discrimination and poverty, as well as traditional norms related to gender [3, 11]. Low literacy levels, low contraceptive up take, the unmet need for family planning in this age group and lack of sexual and reproductive health education to teenagers have also been associated with the high rates of teenage pregnancy [8, 17]. Although the factors contributing to teenage pregnancies have widely been documented, these vary from country to country. Therefore, this study intends to analyze the 2019 Sierra Leone Demographic and Health Survey (SLDHS) to determine the prevalence and factors associated with teenage pregnancy.

## Methods

### Study design

Data used for this secondary analysis was collected during the Sierra Leone Demographic and Health Survey (SLDH) which was a cross sectional survey.

### Data source and sampling procedure

We used the 2019 SLDHS data, which was conducted from May 2019 to August 2019. A stratified two-stage cluster sampling procedure was employed to select study participants. In the first stage, 578 enumeration areas (EAs) (214 urban and 364 rural) were selected leading to 13,872 households. Out of the total weighted sample of 15,574 women in the data set, only 3,427 were adolescents aged 15–19 years. A full protocol with detailed explanation about the data collection process and sampling is available online [10].

### Variables

#### Outcome variable

The outcome variable was teenage pregnancy that included adolescents (15–19 years) who were currently pregnant or had an abortion or had ever given birth were coded as one (1) and zero (0) for those who had never had a pregnancy [19].

#### Independent variables

We included determinants of teenage pregnancy basing on available literature and data collected in this survey [6, 19, 20]. Thirteen independent variables were used and included as shown in Table 1:

**Table 1:** Definition and measurement coding of independent variables

Variable	Survey Question	Coding
Age	In what month and year were you born?	1 = 17–19 2 = 15–16 Note: The individual ages in the dataset were categorized into 17–19 and 15–16
Wealth index	Wealth index (quintiles) was calculated by DHS from responses on household asset ownership using Principal Component Analysis.	1 = poorest quintile 2 = poorer quintile 3 = middle quintile 4 = richer quintile 5 = richest quintile
Residence	-	1 = Urban 2 = Rural
Region	-	1 = Eastern 2 = Northern 3 = Northwestern 4 = Southern 5 = Western
	What is the highest level of school you attended: primary, secondary or higher?	1 = no education 2 = primary education 3 = post-primary education Higher level of education as shown in Table 2 had only 14 adolescents so in the logistic regression analysis, we combined secondary and tertiary into post-primary
Household size	How many people usually live in your household?	1 = less than seven members 2 = seven and above members Note: The categorization was based on the dataset’s average household size
Sex of household head	Is the head of this household male or female?	1 = Female 2 = Male
Working status	What is your occupation? That is, what kind of work do you mainly do?	1 = Yes Working 2 = Not working
Marital status	Are you currently married or living with a man as if married?	1 = Married 2 = Not married Married including those in formal and informal unions
Religion	What is your religion?	1 = Christianity 2 = Islam Note: The dataset had 6 categories; Christianity, Islam, Bahai, Traditional, None and Others. However, all study participants belonged to either Christianity or Islam
Problems seeking permission to get healthcare	Many different factors can prevent women from getting medical advice or treatment for themselves. When you are sick and want to get medical advice or treatment, is each of the following a big problem or not a big problem: a) Getting permission to go to the doctor?	1 = No big problem 2 = big problem
Problems with distance to health facility	Many different factors can prevent women from getting medical advice or treatment for themselves. When you are sick and want to get medical advice or treatment, is each of the following a big problem or not a big problem: b) The distance to the health facility?	1 = No big problem 2 = big problem
Exposure to family planning messages on mass media	In the last few months have you: a) Heard about family planning on the radio? b) Seen anything about family planning on the television? c) Read about family planning in a newspaper or magazine? d) Received a voice or text message about family planning on a mobile phone?	1 = Yes (if exposed to any on television, radio, mobile phone and newspapers). 2 = No (No exposure to any of the 4)

### Statistical analysis

In order to account for the multi-stage cluster study design, we used SPSS version 25.0 statistical software complex samples package incorporating the following variables in the analysis plan to account for the multistage sample design inherent in the DHS dataset: individual sample weight, sample strata for sampling errors/design, and cluster number [21–24]. Analysis was carried out based on the weighted count to account for the unequal probability sampling in different strata and to ensure representativeness of the survey results at the national and regional levels.

Before logistic regression, each independent variable was assessed separately for its association with the outcome variable using bivariable logistic regression and we presented the crude odds ratio (COR), 95% confidence interval (CI) and p-values. Independent variables whose association with teenage pregnancy had a p-value ≤ 0.25 at the bivariable level, and were not strongly collinear (assessed using variance inflation factor cut off at 5) with other independent variables were included in the final multivariable logistic regression model to assess the independent effect of each variable on teenage pregnancy. Adjusted odds ratios (aOR), 95% confidence intervals (CI) and p-values were calculated with statistical significance level set at p-value < 0.05.

## Results

Data of 3,427 female adolescents were included in the analysis. The prevalence of teenage pregnancy was 22.1% [758/3,427]. Of these, 17.8%, (608/3427), had ever had childbirth, 4.2%, (144/3427), were pregnant and 1.2%, (40/3427) had ever terminated a pregnancy. Majority of the adolescents were aged 17 to 19 years (1966/3427), and residents of urban areas (1814/3427). Most of them were Muslims (2620/3427), with at least secondary education (2300/3427), not working (2159/3427) ,and without exposure to family planning messages on mass media (television, radio, newspapers, or mobile phones; 2513/3427). The mean age, and household size were 16.93 ± standard deviation (sd) 1.47 and 7.53 ± sd 3.64 respectively. The details are presented in Tables 2 and 3.

**Table 2:** Socio-demographic characteristics of female adolescents (N = 3427) in Sierra Leone as per the 2019 SLDHS

Characteristics	Frequency (n)	Per cent (%)
**Age**		
15 to 16	1461	42.6
17 to 19	1966	57.4
**Residence**		
Urban	1814	52.9
Rural	1613	47.1
**Region**		
Western	823	24.0
Eastern	648	18.9
Northwestern	560	16.3
Northern	766	22.4
Southern	630	18.4
**Religion**		
Islam	2620	76.5
Christianity	807	23.5
**Household head sex**		
Male	2254	65.8
Female	1173	34.2
**Household Size**		
7 and above	1891	55.2
Less than 7	1536	44.8
**Working status**		
Not working	2159	63.0
Working	1268	37.0
**Education Level**		
No Education	477	13.9
Primary Education	636	18.6
Secondary Education	2300	67.1
Tertiary	14	0.4
**Wealth Index**		
Poorest	434	12.7
Poorer	537	15.7
Middle	682	19.9
Richer	898	26.2
Richest	876	25.6
**Exposure to family planning messages on mass media**		
No	2513	73.3
Yes	914	26.7
**Permission to access healthcare**		
Big problem	871	25.4
Not big problem	2556	74.6
**Distance to health facility**		
Big problem	1429	41.7
Not big problem	1998	58.3
**Marital status**		
Not married	2949	86.1
Married	478	13.9

### SLDH: Sierra Leone Demographic Health Survey

**Table 3:** Prevalence of teenage pregnancy among female adolescents in Sierra Leone

	Frequency N = 3,427	%	95% CI
**Ever had a child**	608	17.8	16.2–18.7
**Currently pregnant**	144	4.2	3.5–4.9
**Ever terminated a pregnancy**	40	1.2	0.8–1.5
**Any of the above**	758	22.1	20.3–23.0

### Factors associated with teenage pregnancy

After adjusting for confounders, (age, wealth index, region, residence, level of education, sex of household head, working status, marital status, religion, exposure to family planning messages on mass media and problems with distance to health facility),the odds of teenage pregnancy among married girls were about 15 times more than the odds among those who were not married (aOR; 15.31, 95% CI: 11.17–20.98) while the odds of teenage pregnancy among girls from the poorest households were 2.5 times more than the odds among girls from the richest households. The details are presented in Table 4.

**Table 4:** Factors associated with teenage pregnancy among adolescents in Sierra Leone as per the 2019 SLDHS

Characteristics	No TP n (%)	Yes, TP n (%)	Crude model cOR (95% CI)	P-value	Adjusted model aOR (95% CI)	P-value
**Marital status**						
Not married	2573(96.4)	376(49.7)	1		1	
Married	96 (3.6)	381 (50.3)	**27.17(20.69–35.69)**	< 0.001	**15.31(11.17–20.98)**	< 0.001
**Age**						
15 to 16	1357 (50.8)	104(13.7)	1		1	
17 to 19	1312 (49.2)	654(86.3)	**6.48 (4.79–8.77)**	< 0.001	**4.33 (3.04–6.16)**	< 0.001
**Residence**						
Rural	1124 (42.1)	488(64.4)	1		1	
Urban	1545 (57.9)	270(35.6)	**0.40 (0.32–0.50)**	< 0.001	0.88 (0.53–1.46)	0.612
**Region**						
Western	709 (26.6)	113(14.9)	1		1	
Southern	442 (16.6)	189(24.9)	**2.68 (1.88–3.82)**	< 0.001	1.37 (0.88–2.14)	0.162
Northwestern	404 (15.1)	156(20.6)	**2.41 (1.65–3.53)**	< 0.001	0.75 (0.45–1.23)	0.250
Northern	623 (23.3)	143(18.9)	1.44 (0.97–2.14)	0.071	0.66 (0.40–1.07)	0.089
Eastern	491 (18.4)	147(20.7)	**2.01 (1.40–2.89)**	< 0.001	1.12 (0.71–1.77)	0.629
**Religion**						
Islam	2024 (75.8)	596(78.6)	1		1	
Christianity	645 (24.2)	161(21.4)	0.85 (0.66–1.11)	0.234	1.02 (0.77–1.36)	0.895
**Household head sex**						
Male	1714 (64.2)	540(71.2)	1		1	
Female	955 (35.8)	218(28.8)	**0.73 (0.60–0.89)**	0.002	1.20 (0.94–1.53)	0.140
**Household Size**						
7 and above	1480 (55.5)	411(54.2)	1		-	
Less than 7	1189 (44.5)	347(45.8)	1.05 (0.86–1.29)	0.637		
**Working status**						
Not working	1816 (68.0)	343(45.3)	1		1	
Working	853 (32.0)	415(54.7)	**2.58 (2.15–3.10)**	< 0.001	1.26 (0.97–1.64)	0.083
**Education Level**						
Post-primary	1918(71.9)	396(52.2)	1		1	
Primary Education	488 (18.2)	148(19.5)	**1.47 (1.16–1.86)**	0.001	1.18 (0.86–1.63)	0.314
No education	263 (9.9)	214(28.3)	**3.93 (3.02–5.11)**	< 0.001	1.43 (0.98–2.08)	0.067
**Wealth Index**						
Richest	782 (29.3)	94 (12.4)	1		1	
Richer	734 (27.5)	164(21.6)	**1.86 (1.32–2.63)**	< 0.001	**1.61 (1.10–2.38)**	0.016
Middle	504 (18.9)	177(23.4)	**2.94 (2.05–4.20)**	< 0.001	**2.22 (1.29–3.80)**	0.004
Poorer	362 (13.6)	175(23.1)	**4.03 (2.81–5.78)**	< 0.001	**2.07 (1.13–3.78)**	0.018
Poorest	287 (10.7)	148(19.5)	**4.32 (2.95–6.32)**	< 0.001	**2.55 (1.31–4.94)**	0.006
**Media exposure**						
No	1923(72.0)	590(77.8)	1		1	
Yes	746 (28.0)	168(22.2)	**0.74 (0.58–0.94)**	0.014	0.95 (0.73–1.24)	0.716
**Permission to access healthcare**						
Big problem	688 (25.0)	203(26.8)	1		-	
Not big problem	2001(75.0)	555(73.2)	0.91 (0.73–1.13)	0.401		
**Distance to health facility**						
Big problem	1055 (39.5)	374(49.3)	1		1	
Not big problem	1614 (60.5)	384(50.7)	**0.67 (0.54–0.83)**	< 0.001	1.02 (0.79–1.32)	0.865

Bold*: significant at < 0.05, cOR; crude odds ratio, aOR; adjusted odds ratio, TP: teenage pregnancy

## Discussion

This study examined the prevalence teenage pregnancy and its associated factors in Sierra Leone using the 2019 SLDHS data. We found that the prevalence of teenage pregnancy was 22.1% and that being married, older (17–19 years) and belonging to the richer, middle, poorer and poorest households were associated with teenage pregnancy. Although the 22% prevalence of teenage pregnancy reported in Sierra Leone here indicates a reduction from the 34% reported between 2008 and 2013 in the country’s 2013 demographic and health survey [10] and the 28% reported in a review by Ahinkorah and colleagues [2], it is still higher than the average of 18.8% prevalence reported by a systematic review across the continent and prevalence in Ethiopia (12.8%) [8, 25]. The lower prevalence in Ethiopia could be partly attributed to the fact that the study did not include adolescents who had ever terminated a pregnancy and only included those who had a child or were pregnant which risked under reporting.

However, despite the prevalence of teenage pregnancy in Sierra Leone being high compared to the average SSA prevalence, the observed decline over the decade (28.3% in 2013 to 22.1% in 2019) is not surprising, because it is in line with the trends over the last decade, which indicate a significant improvement in maternal and child survival indicators in Sierra Leone [12, 26] owing to various strategies by the government and its development partners. For example, in 2018, the government of Sierra Leone developed a national strategy for the reduction of adolescent/teenage pregnancy. The strategy is organized into six pillars including; policy and legal environment, adolescent and young people friendly services, enabling school environments, communication and advocacy, community ownership, and coordination, monitoring, and evaluation [27]. Studies from across the continent show that the reduction in the prevalence of teenage pregnancy is not limited to only Sierra Leone as reports from other sub-Saharan African countries such as Uganda, Ethiopia and Tanzania indicate strides in the fight against teenage pregnancy [23, 28, 29]. This observed reduction in teenage pregnancy prevalence in these countries is majorly attributed to global and governments’ interventions such as promotion of modern contraceptive methods in this age group to prevent unplanned pregnancies [2, 20, 26, 28].

Adolescents who were married, older (17–19 years) and those belonging to poorest, poorer, middle and richer households had more odds of teenage pregnancy compared to the unmarried, younger and those in the richest wealth index respectively. Similar findings have been reported from an analysis of national demographic surveys in Uganda, where the odds of getting pregnant were higher among married teenagers and those who were living with partners as compared to those who had never been married [30]. A systematic review of studies from across Africa, further showed that married/cohabiting/ previously married adolescents were eight times more likely to have first pregnancy compared to never married adolescents [6]. Getting married at an early age is a barrier to schooling and is greatly associated with reduced ability to negotiate for and make own choices on contraceptive use. This makes these girls more vulnerable to continuous unsafe sexual activities that increase their chances of getting pregnant [31]. Other researchers have associated marriage/cohabitation to an increased desire for having children most especially in most sub-Saharan African countries, where adolescent girls may face social pressure to marry and, once married, to have children [6]. All these factors predispose these girls to teenage pregnancy.

Teenage pregnancy was four times more likely among older teenagers (17–19 years) as compared to their younger counterparts. This is in line with findings by a systematic review of studies from across Africa that indicated that the odds of first adolescent pregnancy was higher with increasing age [6]. This may be attributed to a common practice of forcing girls in this age group into early marriages(given that most studies show this age group as the average age of marriage) which exposes them to unsafe sex practices since they are not empowered enough to use contraceptives which increases their chances of getting pregnant [32–34]. Interestingly and uniquely with this analysis, all teenagers, regardless of their wealth quintile had higher odds of teenage pregnancy compared to their counterparts in the richest quintile. This is slightly different from other studies where belonging to the poor wealth quintiles alone was significantly associated with teenage pregnancy [4, 7, 28]. However, even for this study, the odds were higher among those in the poorest and poorer quintiles and less among those in the richer quintiles. Under poverty, most parents often see child labour to engage in remunerative work and early marriage as the viable options for girls, leading to low investment in education [35]. Low education attainment and the effects of early marriage in turn, lead to long-term economic dependency, lower health literacy, limited decision making power, and reduced ability to negotiate for or practice safer reproductive health measures including contraception [36]. Furthermore, adolescent girls from the poor households are likely to have limited financial resources to afford direct and indirect costs involved in accessing contraceptive services [25]. Studies have shown that wealth is associated with access to contraceptive services and knowledge of these services hence, an adolescent girl with economic challenges are more likely to have teenage pregnancy [25, 37].

### Strengths and limitations

This study used the most recent data from the Sierra Leone Demographic Health Survey with a larger sample size and higher quality, which substantially reduces the risk of sampling bias and measurement bias. We also used a nationally representative sample and weighed the data for analysis. Therefore, results from this study can be generalized to all teenagers in Sierra Leone. Furthermore, this study included even girls that had ever terminated a pregnancy which ensured a reduced risk of under reporting since most studies only report prevalence basing on teenagers who have given birth or who are pregnant. Most data were self-reported and could not be verified through records which carries a risk of social desirability bias. Finally, data on the independent variables in this analysis refer to the time of the survey, and may differ to the experience at the time of pregnancy.

## Conclusion

This study reveals that the prevalence of teenage pregnancy is high, and associated with being married, older with no discrimination in the wealth quantile status of the teenagers. Policies giving teenage mothers a second chance by encouraging them to return to school after childbirth should be encouraged as an alternative to early marriages. Provision of adolescent-friendly health services in schools and health facilities should be strengthened targeting mainly the older adolescents and those from poor households with reproductive health counselling including contraceptive services availability.

## Data Availability

The data set used is openly available upon permission from MEASURE DHS website (URL: https://www.dhsprogram.com/data/available-datasets.cfm). However, authors are not authorized to share this data set to the public but anyone interested in the data set can seek it with written permission from MEASURE DHS website.

## Abbreviations

EA: Enumeration area
AOR: Adjusted Odds Ratio
CI: Confidence Interval
COR: Crude Odds Ratio
DHS: Demographic Health Survey
SLDHS: Sierra Leone Demographic Health Survey
OR: Odds Ratio
SD: Standard Deviation
WHO: World Health Organization
SPSS: Statistical Package for Social Science
